# Low *RCAN1.2* mRNA Expression Is Associated with Poor Prognosis of Patients with Esophageal Squamous Cell Carcinoma

**DOI:** 10.7150/jca.84307

**Published:** 2023-07-31

**Authors:** Haijun Yang, Jiahuan Zhou, Keyao He, Junkuo Li, Fang Zhao, Ningtao Dai, Shouxin Wu, Wushuang Li, Jiangman Zhao, Yaowen Zhang, Fuyou Zhou

**Affiliations:** 1 Anyang Tumor Hospital, The Affiliated Anyang Tumor Hospital of Henan University of Science and Technology, Anyang 455000, Henan, China.; 2 Henan Key Medical Laboratory of Precise Prevention and Treatment of Esophageal Cancer, Anyang 455000, Henan, China.; 3 Shanghai Zhangjiang Institue of Medical Innovation, Shanghai Biotecan Pharmaceuticals Co., Ltd., Shanghai 201204, China.; 4 The Third Affiliated Hospital of Xinxiang Medical University, Xinxiang 453003, Henan Province, China.

**Keywords:** *RCAN1*, esophageal cancer, tumor suppressor, prognosis, biomarker

## Abstract

**Background:** This study aims to explore the role of *RCAN1* in esophageal squamous cell carcinoma (ESCC) cells, determine the mRNA level of three *RCAN1* isoforms in ESCC tissue, and evaluate the prognostic value of three *RCAN1* isoforms.

**Methods:** Colony-forming assay, Wound-healing assay and Transwell assay were used to evaluate the effect of *RCAN1* on cell proliferation, migration and invasion. The mRNA expression of three *RCAN1* isoforms was detected in paired tumor and normal tissues from 100 ESCC patients by real-time PCR. Kaplan-Meier survival curves and Cox proportional hazards model were used to evaluate the prognostic value of three *RCAN1* isoforms. A nomogram was used to predict the probability of 2-year and 5-year overall survival (OS).

**Results:** In vitro, knockdown of *RCAN1* could promote ESCC cell proliferation, migration and invasion abilities. Compared to the paired normal tissues, *RCAN1* isoform 1 (*RCAN1.1*, *P*=0.0027) and *RCAN1* isoform 2 (*RCAN1.2*, *P*=0.0006) were significantly decreased in tumor tissues. The low expression of *RCAN1.2* mRNA was associated with advanced stage (*P*=0.0176) and lymph node metastasis (LNM, *P*=0.0219). ESCC patients with low *RCAN1.2* mRNA levels had shorter survival time compared to those with high *RCAN1.2* levels (*P*=0.007). Multivariate COX analysis indicated that *RCAN1.2* mRNA level was an independent prognostic indicator of OS of patients with ESCC (hazard ratio=0.5266, *P*=0.03554). The concordance index of nomogram to predict OS was 0.693 based on LNM, *RCAN1.2*, tumor stage and patients' age.

**Conclusion:** These findings show that *RCAN1* gene play a role in preventing proliferation, migration, and invasive activity of ESCC cells. *RCAN1.2* mRNA level is a novel prognostic marker in ESCC, targeting *RCAN1.2* may provide a potential therapeutic approach in ESCC.

## Introduction

Esophageal cancer (EC) is one of the leading causes of cancer-related mortality worldwide. Together with other types of gastrointestinal cancers, esophageal cancer causes almost a third of disability-adjusted life-years (DALYs) due to cancer 1. Although great efforts have been made to improve the treatment strategy, the prognosis of EC patients remains poor, with a low 5-year survival rate 2. It is considered that lack of valid biomarker for early diagnosis and efficacious drugs are the main causes 2, 3. Therefore, it is vital to further explore the pathogenic process that leads to EC, identify novel molecular markers or causative genes, so as to improve the prognosis of EC and search for new drug therapeutic target.

Regulator of calcineurin 1 (*RCAN1*, also known as Down's syndrome critical region 1 [*DSCR1*]) is one of the genes located on chromosome 21 which was considered as a tumor suppressor in various cancers 4. There are three main transcripts of *RCAN1* (*RCAN1.1, RCAN1.2,* and *RCAN1.4*) detected in human tissues 5. Previous studies showed that upregulation of* RCAN1* could inhibit *VEGF*-mediated proliferation of endothelial cells and suppress tumor angiogenesis 4, 6. In addition, overexpression of *RCAN1* could reduce cell viability in lymphoma Raji cells and restrain the lymphoma growth in mice 7. In some gastrointestinal cancers, for instance, it was reported that *RCAN1* had suppressive effect on transformed properties of early stage colorectal cancer cells 8. Meanwhile, overexpression of *RCAN1.4* in HCC cells could prevent tumor growth, migration, and angiogenesis and metastases 9. While the role of *RCAN1* in esophageal cancer is still unknown.

Given that over half of all EC-related deaths occur in China where approximately 90% of esophageal cancer cases are esophageal squamous cell carcinoma (ESCC), particularly in high-risk populations^10^. In the present study, we aimed to explore the function of *RCAN1* in the proliferation, migration and invasion of ESCC cells, and to determine the mRNA level of three *RCAN1* isoforms in ESCC tissue, compared with adjacent normal tissues. The results of this study may give a novel insight in the role of *RCAN1* in ESCC pathogenesis and suggested that *RCAN1.2* mRNA level is a potential prognostic biomarker.

## Materials and methods

### Patients and samples

In this study, a total of 100 ESCC patients with complete clinicopathological data were enrolled in the Anyang Tumor Hospital from 2013 to 2018. The inclusion criteria for participants in this study were (1) Newly diagnosed ESCC confirmed by postoperative histology, (2) receiving surgery resection and with negative resection margin. Exclusion criteria were (1) use of preoperative neoadjuvant therapy, (2) with other malignancies, (3) loss of follow-up. 100 pairs of primary tumor tissues and adjacent normal tissues were collected for real-time PCR. The clinical characteristics of 100 ESCC patients were showed in **Table 1**.

This research was approved by the Ethical Review Committee of Anyang Tumor Hospital and informed consent was obtained from all participants. All procedures performed in this study were in accordance with the ethical standards of the institutional and/or national research committee and with the Helsinki declaration.

### Cell culture and transfection

Esophageal squamous cell carcinoma cell line KYSE30 was obtained from the Shanghai Bogu Biological Cell Institute (Shanghai, China). ESCC cells were cultured in RPMI-1640 medium containing 10% fetal bovine serum (FBS; all from Gibco, Thermo Fisher Scientific, USA) at 37℃ in a humidified atmosphere with 5% CO_2_. Lentivirus-knockdown *RCAN1* particles (Lenti-KD), knockdown control particles (Lenti-ctrl) were purchased from FulenGen Co., Ltd. (Guangzhou, China). For infections, KYSE30 cells were incubated with lentiviral particles and Polybrene (1 μg/ml) in RPMI-1640 medium. After 12 h, the infection medium was replaced by fresh culture medium. After 48 hours of transfection, cells were used for subsequent experiments.

### Western blotting

Protein homogenates were separated by 10% SDS-PAGE electrophoresis and were then transferred to a PVDF membrane (Millipore, USA). PVDF membrane was blocked with 5% skim milk for 1 h at room temperature (RT). Primary antibody anti-RCAN1 (1:1000, Abcam, USA) and rabbit anti-human GAPDH polyclonal antibody (1:2000, Kangwei, Shanghai, China) were added and incubated at 4°C overnight. The membrane was washed 3 times for 5 min each time with TPBS, and then the secondary antibodies (goat-anti-rabbit, 1:2000, Kangwei, Shanghai, China) were incubated at RT for 1 h. The membrane was washed 3 times with TPBS. The signal was visualized by chemiluminescence and the relative expression levels were analyzed by Bio-Rad molecular imaging system (Hercules, CA).

### Colony-forming assay

A total of 500 cells per well were seeded in 6-well plates and were cultivated in complete media for 7-14 days. Subsequently, media was removed, cells were washed twice in PBS. Cells were then fixed by 4% paraformaldehyde (Sigma-Aldrich, Germany) for 1h and stained with 0.5% crystal violet (Sigma-Aldrich, Germany) for 30 min at RT. Plates were thoroughly washed with water and air-dried at RT.

### Wound-healing assay

For the wound healing assay, KYSE30 cells were inoculated in 6-well plates and form a confluence cell monolayer for about 12h. Each group had three wells. A pipette tip (200 μl) was used to scratch the culture well, then the wells were washed three times with PBS to remove the floating cells. Cells were cultured in serum-free medium in a humidified incubator (37˚C, 5% CO_2_), and were observed and captured every 3 hours, until 24 h post-scratching. The percentage of wound closure was calculated by the formula: wound closure = (original gap distance - gap distance at the indicated time)/original gap distance × 100%.

### Transwell assay

The invasion assay was evaluated using the 24-well Transwell chambers (Corning, Germany). The chambers were precoated with Matrigel (Corning, Germany). The transfected cells (cell density of 1×106 cells) with serum-free medium were seeded into the upper chamber of each well. The lower compartment was filled up with culture medium containing 15% FBS as the chemoattractant. After incubation at 37℃ for 24 hours, the cells attached to the lower chambers were stained with 0.1% crystal violet after fixation and counted under a light microscope (Nikon, Japan).

### RNA extraction and quantitative real-time PCR

Total mRNAs were extracted from tissues by TRIzol reagent (Invitrogen, USA) following the manufacturer's manual. Reverse transcription was carried out using PrimeScript RT reagent kit (TaKaRa, Japan). Quantitative real-time polymerase chain reaction (qRT-PCR) was then performed by using a 7500 Real-Time PCR System (Applied Biosystems, USA) with the SYBR green I Master Mix Kit (Invitrogen, USA). *GAPDH* was used as an internal control gene, and the relative expression data were normalized to *GAPDH*. All expression data were quantified using the 2^-ΔΔCt^ method. The 2^-ΔΔCt^ value lower than median value of 100 tumor samples was defined as Low expression level, higher than median value as high expression level. The sequences of primers of *RCAN1.1, RCAN1.2* and *RCAN1.4* for qPCR were listed in **Table S1.**

### Follow up

The patients were followed up from first time treatment to August 2022. After hospital discharge, the patients were followed-up regularly every 3 months during the first 2 years, every 6 months from year 3 to 5, and once a year afterwards. Overall survival (OS) was defined as the time from first time treatment to death or final follow-up. During the follow-up period, 52 ESCC patients reached the end point of follow-up, their median OS was 24.5 months (range 2-92 months). Forty-eight patients were currently alive.

### Expression level analysis of three *RCAN1* transcripts in external cohorts

Two datasets (GSE164158 and GSE149609) were downloaded from Gene Expression Omnibus (GEO) database to validate the mRNA expression level of three *RCAN1* transcripts in external cohorts. After quality control and reads mapping^11^, Fragments Per Kilobase of transcript per Million fragments mapped (FPKM) and reads count of all transcripts of *RCAN1* were calculated to quantify its expression level using StringTie software^12^. DESeq2 package^13^ in R was used to identify *RCAN1* transcripts' expressional difference between tumor and matched normal tissues.

### Statistical analysis

Data was analyzed using IBM SPSS Statistics 22 software (IBM, USA) and GraphPad Prism 6.0 software (GraphPad Software, CA) and R project. The difference in distribution of categorical variables between groups were analyzed by Chi-square or the Fisher's exact test. For continuous variables, the significant differences between groups were assessed using Student t test for normal distribution data, the Mann-Whitney U test for non-normal distribution data. Kaplan-Meier analysis was used to perform survival analysis with log-rank test to compare the differences between subgroups. Then, propensity score matching (PSM) method by MatchIt package in R was used to remove confounding bias of clinical factors associated with prognosis. A nomogram was established using prognostic factors determined by Cox proportional hazards model to predict patients' 2 and 5-year survival probability. Internal validation by the bootstrap method was performed, the concordance index (C-index) and calibration curve were calculated to assess the predictive accuracy and discriminatory capacity. The nomogram was generated using R 4.0.2 and third-party package *rms* and its auxiliary program package*.* The *P* value of less than 0.05 was considered statistically significant.

## Results

### Effect of *RCAN1* downregulation on ESCC cell proliferation, migration, and invasion

**Figure 1:**
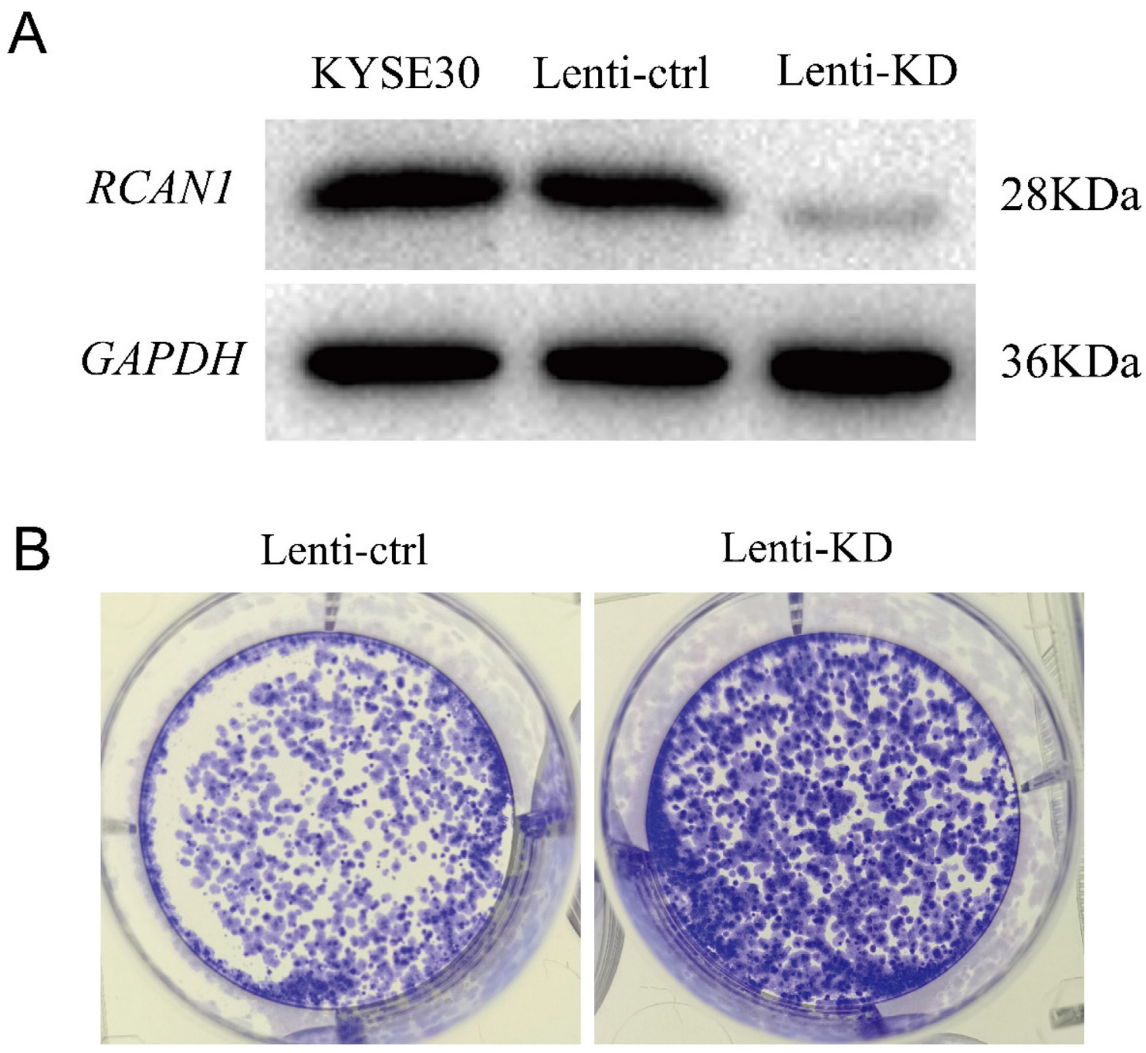
Effect of *RCAN1* downregulation on ESCC Cell Proliferation, Migration, and Invasion. **(A)** The effect of *RCAN1*-targeting siRNAs in KYSE30 cells was confirmed by Western blotting analysis. **(B)** Knockdown *RCAN1* could significantly increase the colony formation in soft agar.

**Figure 2:**
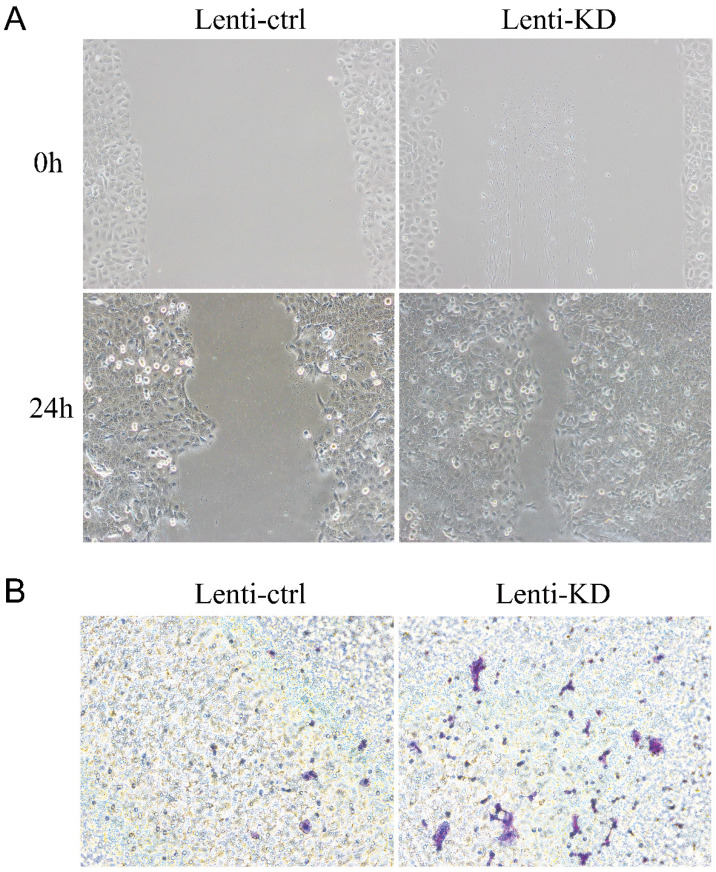
**(A)** *RCAN1* deletion dramatically promoted the migratory ability of ESCC cells in a wound-healing assay. **(B)** Downregulation of *RCAN1* in ESCC cells facilitated cell invasion.

Firstly, we explored the functional role of *RCAN1* in ESCC progression. We established stable model of *RCAN1* knockdown by cell transfection with *RCAN1* shRNA in KYSE30 cells. The result in **Figure 1A** showed that *RCAN1* expression was successfully downregulated in the ESCC cell line. The colony-forming assay results revealed that knockdown of *RCAN1* could promote ESCC cell proliferation (**Figure 1B**). The migration and invasion were evaluated by wound-healing assay and Transwell assay, respectively. The results showed that ESCC cell migration and invasion abilities also were improved by the downregulation of *RCAN1* (**Figure 2A, 2B**).

### Decreased mRNA expression of *RCAN1.1* and *RCAN1.2* in tumor tissues of ESCC

**Figure 3:**
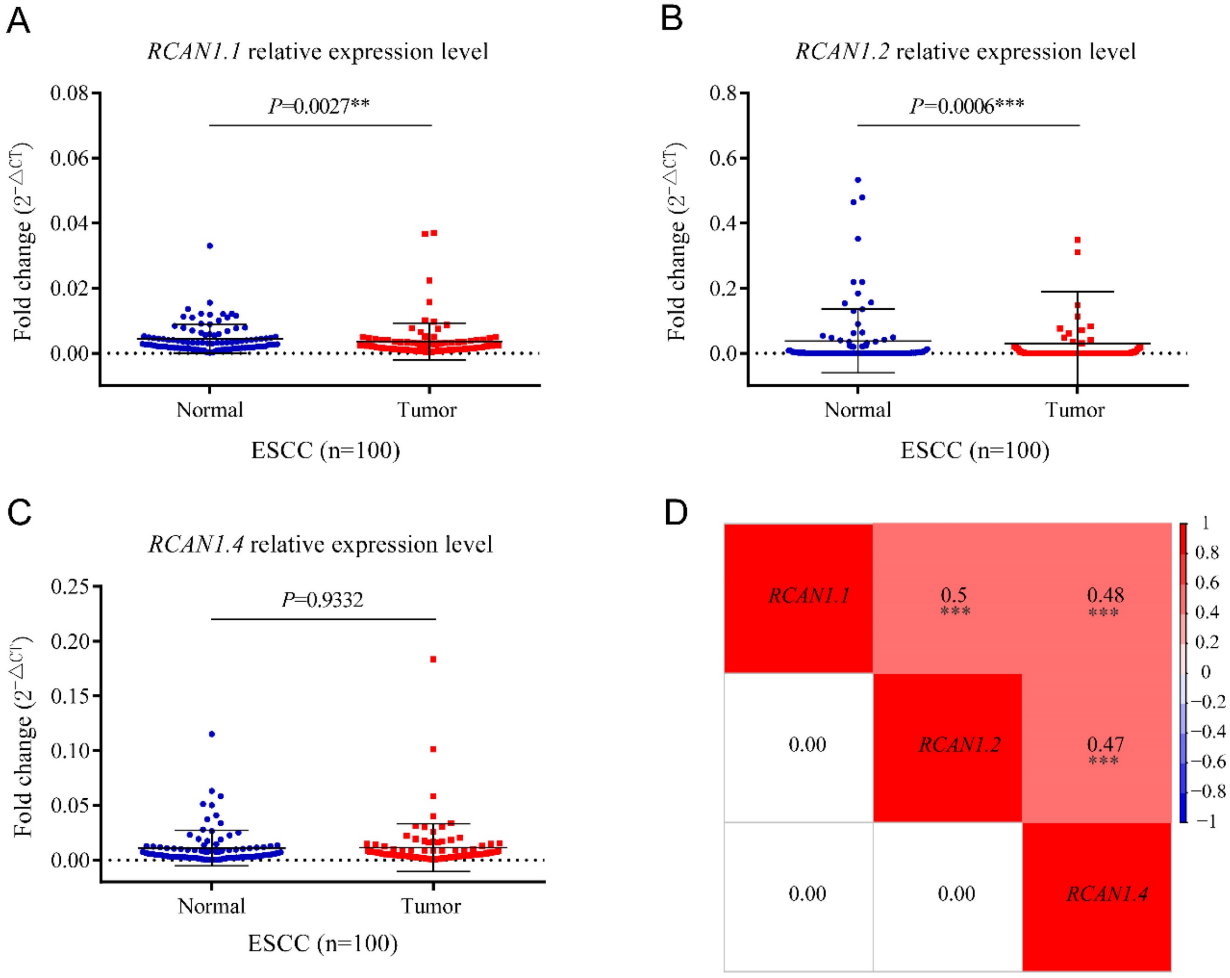
mRNA levels of *RCAN1.1* **(A)**, *RCAN1.2* **(B)** and *RCAN1.4* **(C)** transcripts were determined by qRT-PCR in tumor tissues and matched adjacent normal tissues of esophageal cancer patients. Line and error bar represent mean ± SD; **P < 0.01, ***P<0.001. **(D)** Heatmap of correlation among *RCAN1.1, RCAN1.2* and *RCAN1.4* relative mRNA expression levels in tumor tissues of ESCC patients. The value in the grids of the upper triangle is Spearman correlation coefficient (Spearman r), which is marked by colors (red: positive correlation; blue: negative correlation). The value in grids of the lower triangle is P value of Spearman correlation. ∗∗∗P < 0.001.

To determine the expression of three isoforms of *RCAN1* (*RCAN1.1, RCAN1.2*, and *RCAN1.4*) in clinical samples, we detected the three *RCAN1* isoforms in 100 pairs of ESCC and adjacent noncancerous tissues using real-time PCR. Transcript *RCAN1.1* (*P*=0.0027, **Figure 3A**) and *RCAN1.2* (*P*=0.0006, **Figure 3B**) were significantly decreased in tumor tissues compared to their corresponding tumor-adjacent tissues. However, *RCAN1.4* displayed no significant difference (*P*=0.9332, **Figure 3C**). To further explore the relationship among three isoforms of *RCAN1*, we performed Spearman correlation analysis among relative expression levels of *RCAN1.1, RCAN1.2* and *RCAN1.4*. The results indicated any two *RCAN1* isoforms' expression level was significantly and positively related (**Figure 3D**).

In external cohorts, *RCAN1.2* was down-regulated in ESCC tumor tissues compared with matched normal tissues in both two external cohorts (**Table S2**). Although without significant difference, the expression trend of *RCAN1.2* in external cohorts was consistent with that in our cohort, which accumulated evidences to verify the results about *RCAN1.2* in our study. In addition, the expression trend of *RCAN1.4* was contradictory in GSE164158 and GSE149609 datasets without significance, which from a side proved our results. The *RCAN1.1* expression trend was also contradictory in GSE164158 and GSE149609 datasets without significance, but significantly decreased in our cohort, which needs further investigation.

### Correlation between *RCAN1.1, RCAN1.2* and *RCAN1.4* mRNA expression and clinical characteristics of patients with ESCC

**Figure 4:**
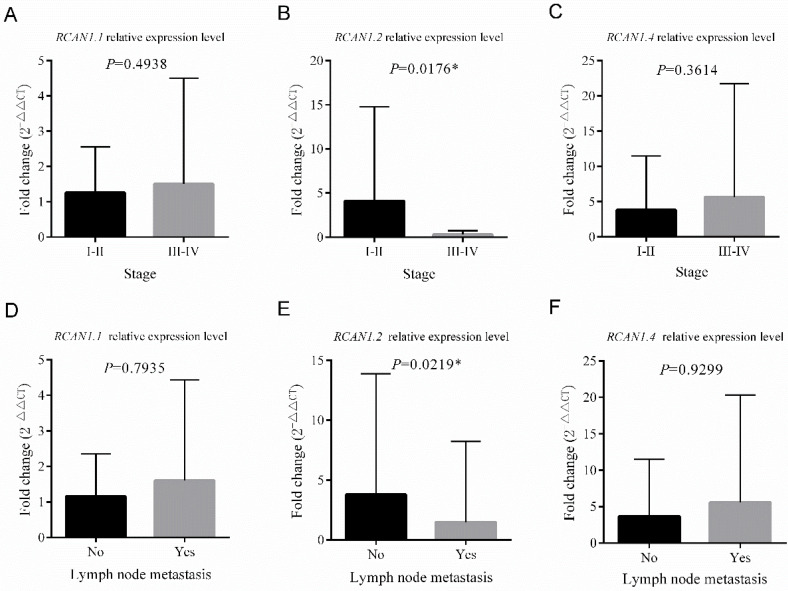
Comparison of the relative mRNA expression levels of *RCAN1.1, RCAN1.2* and *RCAN1.4* according to Stage **(A-C)** and Lymph node metastasis** (D-E)** in 100 esophageal cancer tissues. Line and error bar represent mean ± SD; *P < 0.05.

**Figure 5:**
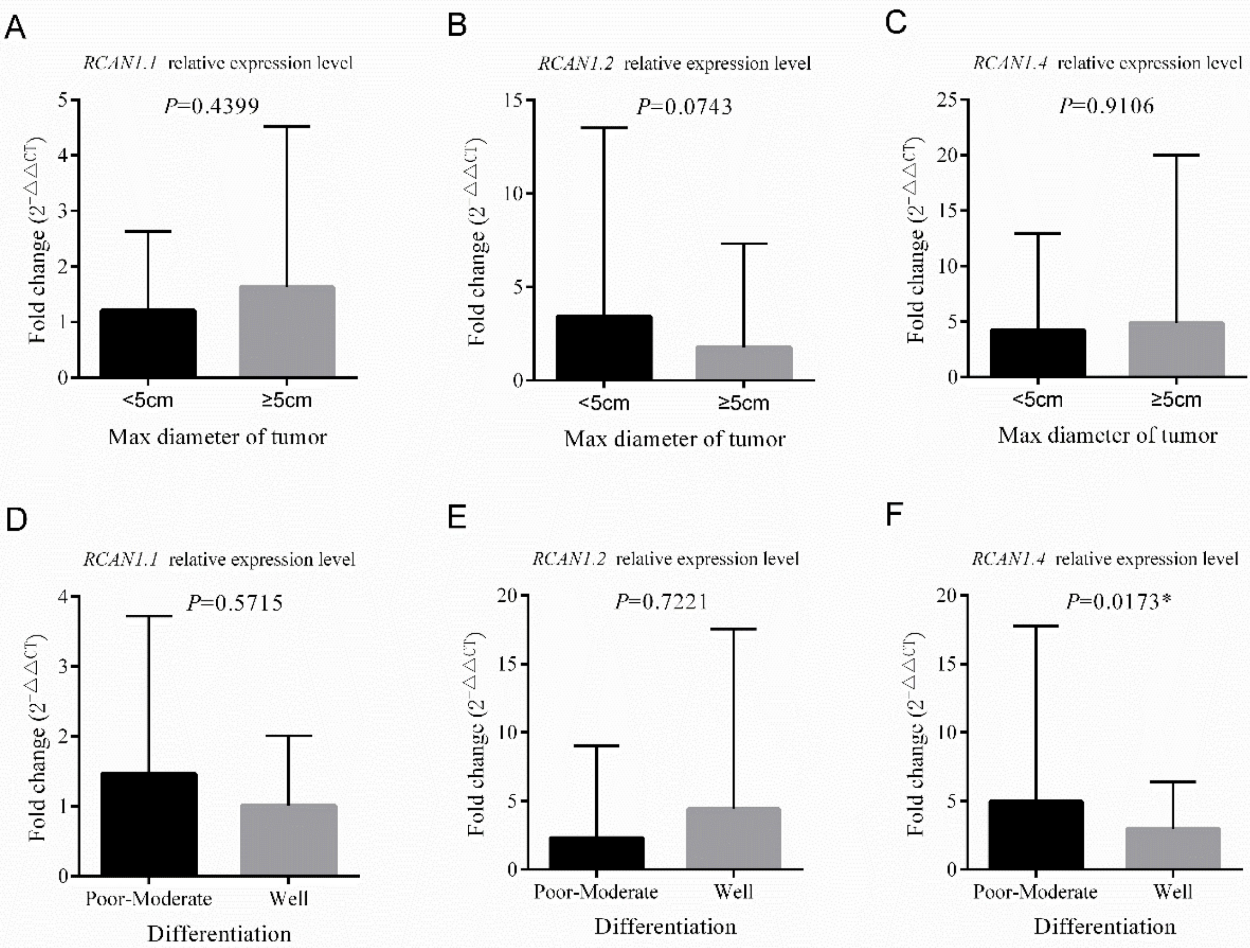
Comparison of the relative mRNA expression levels of *RCAN1.1, RCAN1.2* and *RCAN1.4* according to Max diameter of tumor **(A-C)** and Differentiation of tumor** (D-E)** in 100 esophageal cancer tissues. Line and error bar represent mean ± SD; *P < 0.05.

The patients were divided into low expression group (n=50) and high expression group (n =50) based on the median mRNA expression value of three *RCAN1* isoforms in ESCC tissues. The analysis of the relationship between the mRNA expression of *RCAN1.2* and clinical pathological features (**Table 1**) revealed that the low mRNA expression of *RCAN1.2* in ESCC tissues was significantly associated with male sex (*P*=0.023), advanced stage (*P*=0.032), and LNM (*P*=0.008). No significant association was found between *RCAN1.2* level and age, drink history, family history of cancer, concomitant cardiovascular and cerebrovascular diseases, and differentiation (all *P* > 0.05). Although without significance, the tumors with large size (max diameter≥5cm, *P*=0.052) and patients with smoke history (*P*=0.069,) had much high ratio of low *RCAN1.2* level. **Figure 4** showed, in patients with advanced stage (III-IV), the mRNA expression of *RCAN1.2* was significantly lower than that in patients with early stage (*P*=0.0176, **Figure 4B**). And the mRNA expression of *RCAN1.2* was significantly lower in patients with LNM than that in patients without LNM (*P*=0.0219, **Figure 4E**). These results indicated that *RCAN1.2* might be involved in the development and progression of ESCC. While no significant difference in *RCAN1.2* mRNA expression was found between patients with different tumor size (*P*=0.0743, **Figure 5 B**) and tumor differentiation (*P*=0.7221, **Figure 4E**).

**Table S3** showed low *RCAN1.1* (*P*=0.015) expression were related with younger age (*P*=0.015). There was no association between *RCAN1.1* expression level and other clinical features including tumor's stage, LNM, size, differentiation, gender, smoke and drink history, family history of cancer, and concomitant cardiovascular and cerebrovascular diseases (**Figure 4-5** and **Table S3**). **Table S4** revealed low *RCAN1.4* was significantly associated with younger age (*P*=0.015), drink history (*P*=0.015), and poor-moderate differentiation (*P*=0.026). However, **Figure 5F** showed the *RCAN1.4* expression level was higher in tumors with poor-moderate differentiation than that in tumors with well differentiation.

### *RCAN1.2* as an independent prognosis factor in ESCC

**Figure 6:**
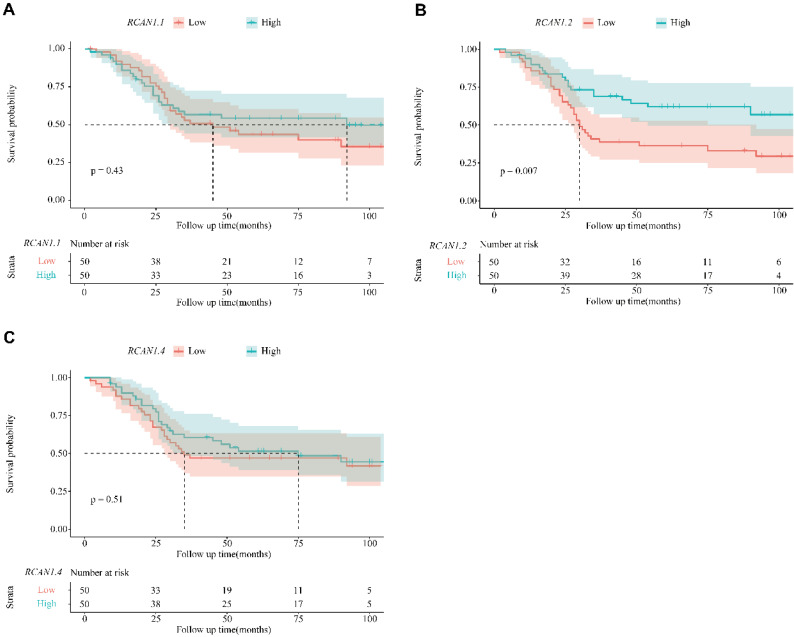
Kaplan-Meier overall survival curve for patients with esophagus cancer between the two groups of *RCAN1.1* high and *RCAN1.1* low mRNA expression** (A)**, *RCAN1.2* high and *RCAN1.2* low mRNA expression** (B),** *RCAN1.4* high and *RCAN1.4* low mRNA expression **(C)**.

**Figure 7:**
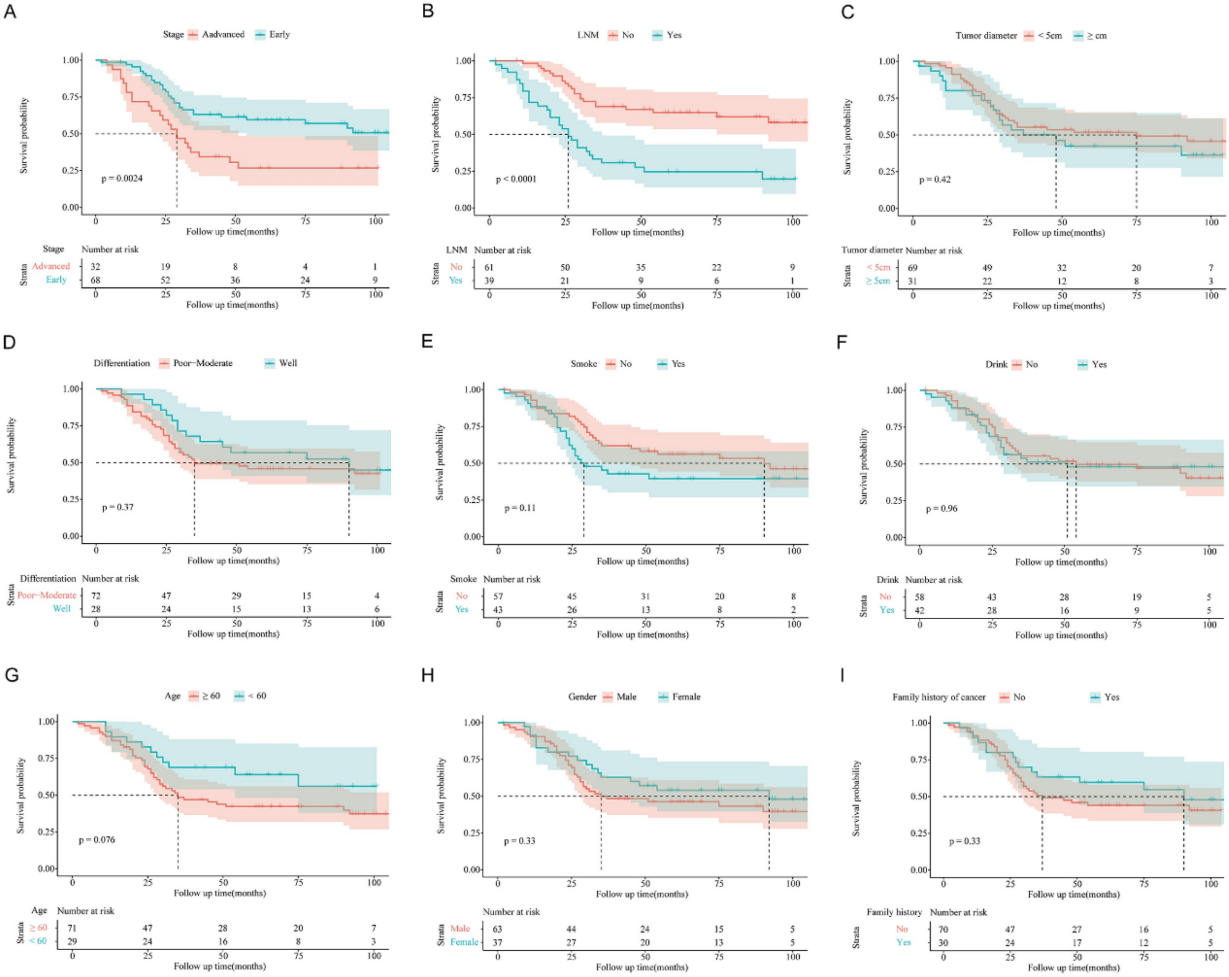
Kaplan-Meier overall survival curve for patients with esophagus cancer according to stage** (A)**, lymph node metastasis status **(B)**, tumor diameter **(C)**, differentiation **(D)**, smoke history **(E)**, drink history **(F)**, age **(G)**, gender** (H)** and family history of caner** (I).**

The Kaplan-Meier survival curves according to *RCAN1.1, RCAN1.2* and *RCAN1.4* mRNA expression level were shown in **Figure 6,** which uncovered that ESCC patients with low *RCAN1.2* mRNA levels had shorter survival time compared to those with high *RCAN1.2* mRNA levels (*P*=0.007, **Figure 6B**). Furthermore, the subsequent Cox's univariate and multivariate analysis (**Table 2**) demonstrated that *RCAN1.2* mRNA level was an independent prognostic indicator of postoperative OS of patients with ESCC (high vs low expression, hazard ratio=0.5266, *P*=0.03554). However, there was no significant influence on patients' OS of *RCAN1.1* and *RCAN1.4* expression level (**Figure 6** and **Table 2**). The Kaplan-Meier survival curves according to clinical features were shown in **Figure 7.** As expected, patients with advanced stage (**Figure 7A**, *P*=0.0024) and LNM (**Figure 7B**, *P*<0.0001) had shorter OS time. Multivariate Cox analysis showed LNM was independent risk factor of poor prognosis (hazard ratio=4.1575, *P*=0.00112, **Table 2**).

**Figure 8:**
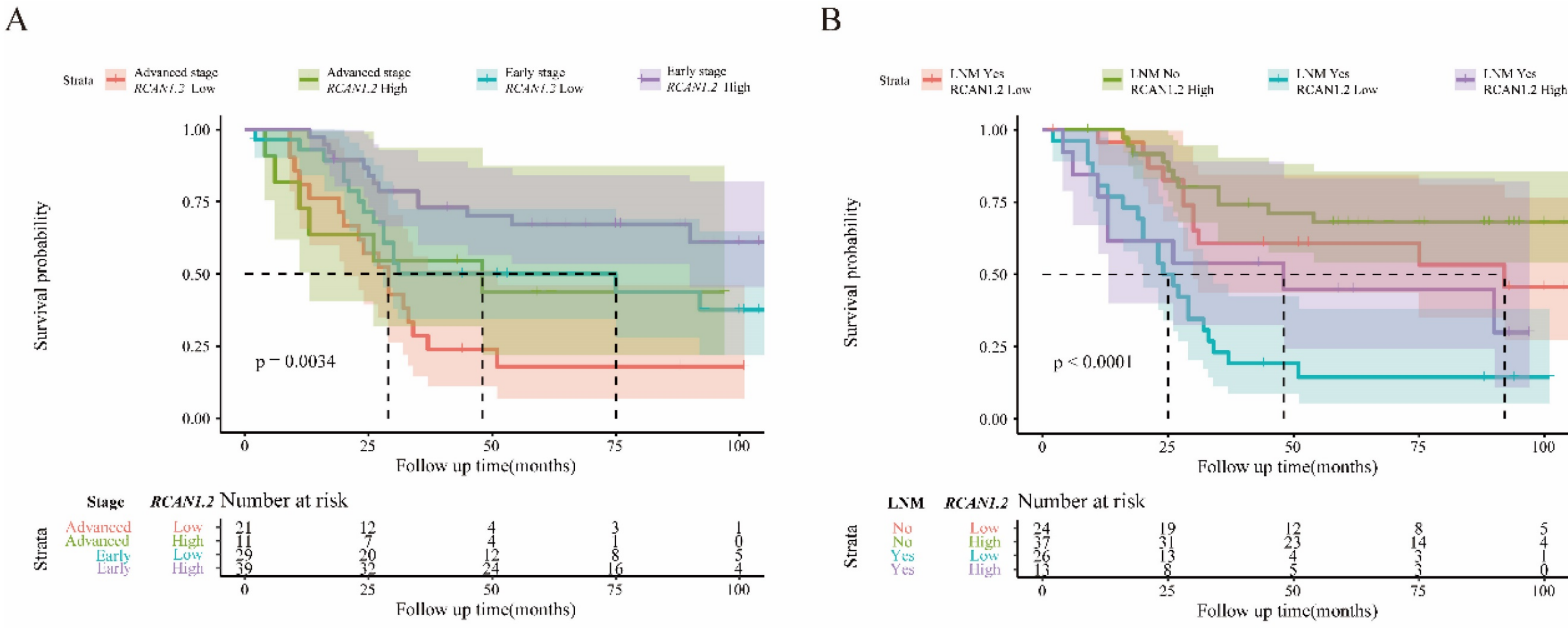
** (A)** Kaplan-Meier overall survival curve for patients with esophagus cancer according to tumor stage and *RCAN1.2* mRNA level.** (B)** Kaplan-Meier overall survival curve for patients with esophagus cancer according to LNM status and *RCAN1.2* mRNA level.

To further determine the intrinsic impact of *RCAN1.2* mRNA level on prognosis, we performed PSM analysis to make tumor stage and LNM status distribution comparable between low *RCAN1.2* group and high *RCAN1.2* group. A total of 87 ESCC patients were selected, including 50 patients with low RCAN1.2 level and 37 patients with high RCAN1.2 level. The results of Cox proportional hazards analysis after PSM analysis were shown in **Table S5**. From **Table S5**, we can see low *RCAN.2* level is still a risk factor of poor prognosis. However, its independent predictive power has been weakened (*P*=0.05737) after rigorous adjustment by tumor stage and LNM status, combining PSM analysis with Multivariate COX analysis. The results in **Figure 8** indicated *RCAN1.2* mRNA level could furtherly refine patients' prognosis based on tumor stage and LNM status.

### A nomogram to predict survival probability of ESCC patients

**Figure 9:**
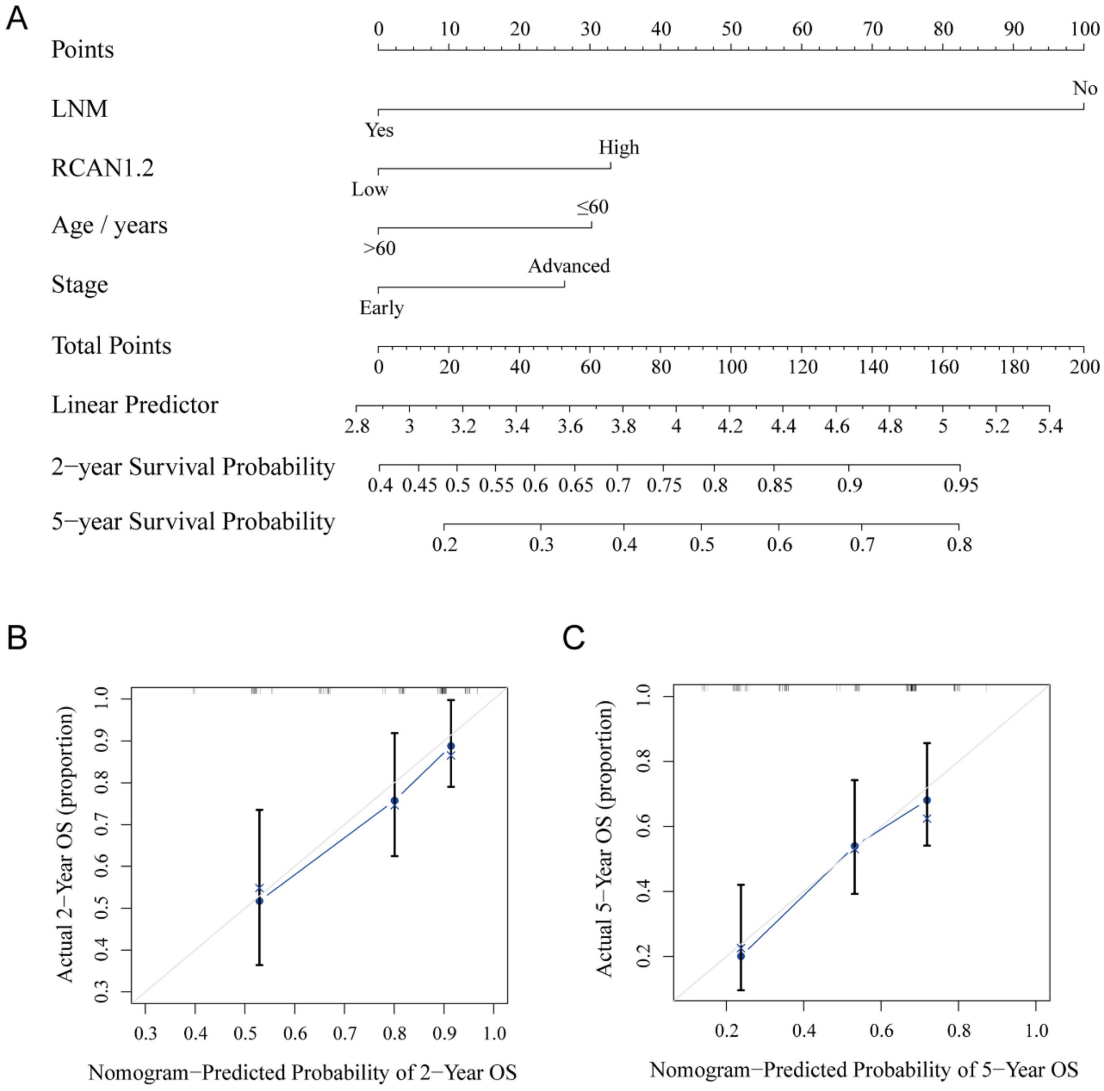
**(A)** A nomogram predicting the 2-year survival probability and 5-year survival probability based on lymph node metastasis status, *RCAN1.2* relative mRNA expression level, patients' age and tumor stage. **(B-C)** The calibration curves for the nomogram to predict 2-year survival probability** (B)** and 5-year survival probability **(C)**.

Nomogram was constructed based on Cox regression model with R. After univariable analysis, the variables of *P*<0.1 including *RCAN1.2*, LNM, stage and age were entered into build nomogram. A total score was calculated to predict 2-year and 5-year survival probability of ESCC patients, which was shown in **Figure 9A**. The C-index and calibration curve were used to evaluate the predictive performance of the nomogram. The C-index of the nomogram was 0.693. The calibration curves of the nomogram were presented in **Figure 9B** and **9C**, which showed the probability of 2-year and 5-year survival were well consistent between the predictive and actual observed survival rates.

## Discussion

Esophageal squamous cell carcinoma is the predominant histologic subtype of esophageal cancer 14. Its fast growth rate and high probability of regional and distant metastasis contributes to the poor prognosis 15. ESCC was associated with a poorer survival compared with esophageal adenocarcinoma (EAC) 16. Evidences showed that there are distinct molecular differences between squamous cell carcinomas and adenocarcinomas 17, 18, implying different signaling pathways involved in the development and progression of esophageal cancer with different subtypes. In this study, we found that downregulation of *RCAN1* had a tumor-promoting effect on ESCC cells and *RCAN1.2* mRNA level of tumor tissue may be as predictor for ESCC postoperative prognosis.

The *RCAN1* gene, located on chromosome 21 in the Down syndrome critical region, has been reported to participate in a wide range of biological process and various pathophysiological changes, including Down syndrome, Alzheimer's disease and type 2 diabetes^19^. Human tissues express three main *RCAN1* transcript isoforms (*RCAN1.1*, *RCAN1.2* and *RCAN1.4*), generated via alternatively splicing. Previous studies showed that *RCAN1* and its isoforms play a critical role on tumor inhibition. For example, in oral squamous cell carcinomas (OSCC), silencing *RCAN1* could reverse the cell proliferative inhibition, cell cycle arrest and cell apoptosis induced by miR-103a-3p knockdown^20^. And *RCAN1.4* knockdown could promote cell proliferation, migration and invasion, as well as improved sunitinib resistance in clear cell renal cell carcinoma 21. Additionally, suppression of *RCAN1.4* induced cell proliferation and metastasis in NSCLC cells 22 and thyroid cancer cells 23. In our current study, we found that *RCAN1* downregulation enhanced ESCC cells proliferation, migration and invasion. These results indicated that *RCAN1* may serve as a tumor suppressor in ESCC.

*RCAN1* was previously shown to be a competitive inhibitor of phosphatase calcineurin, leading to restraint of nuclear factor of activated T cells (NFAT) activation and signaling axis^24^. Several findings have demonstrated that calcineurin-NFAT pathway plays an important role on malignancy and tumor progression 25-27. It was reported that the activation of c-Myc, which is a critical transcription factor for cancer cell proliferation was regulated by activated NFAT binding to an element located in the minimal c-Myc promoter 28. Previous study showed that *RCAN1.4* could prevent proliferation, migration, and invasive activity of hepatocellular carcinoma by reducing calcineurin activity and blocking nuclear translocation of NFAT 9. Meanwhile, *RCAN1* was found as an endogenous inhibitor of NF-κB signaling pathway through interacting with IκBα and affect the phosphorylation of IκBα at tyrosine 42. Overexpression of *RCAN1* could restrain the lymphoma growth in mice via inhibiting NF-κB^7^. In addition, the transcription of cyclo-oxygenase 2 (COX2) was also induced by NFAT proteins. COX2 has been uncovered as a key enzyme in tumor cell migration and the metastatic dissemination of most human tumors^29^. It has been reported that *RCAN1* might act as endogenous negative modulator of COX-2 expression and activity by inhibiting calcineurin and NF-кB pathways to maintain vascular contractility and stiffness^30^. In the present study, we found that knock-down *RCAN1* in ESCC cell could improve cell migration and invasion abilities. However, the potential mechanisms of *RCAN1* in ESCC need further exploration.

Besides *RCAN1* plays a role in the tumor cell proliferation, migration and invasion. Previous studies have demonstrated that *RCAN1* is involved in angiogenesis. For instance, *RCAN1.4* regulates endothelial cell migration by establishing endothelial cell polarity in response to VEGF 31. Meanwhile, VEGFA play a vital role in *RCAN1.4*-mediated PDAC angiogenesis^32^. Moreover, *RCAN1.4* could regulate epithelial-mesenchymal transition (EMT) in sunitinib-resistant clear cell renal carcinoma cell lines 21. In this study, decreased *RCAN1.2* mRNA expression was found in tumor tissues compared with adjacent tissues. However, the role of *RCAN1.2* in cancer progression is barely reported. Our results suggested that the mRNA expression of *RCAN1.2* in ESCC tissues was associated with tumor stage and LNM. And *RCAN1.2* is an independent prognosis factor in ESCC. A nomogram based on *RCAN1.2* expression, tumor stage, LNM and patients' age presented good performance to predict 2-year and 5-year survival probability.

Despite the novelties in this study, there are some limitations worthy of statement. We firstly confirmed *RCAN1*'s functional role in vitro, then performed clinical study to identify *RCAN1.2* mRNA as an independent prognosis factor of ESCC. Because commercialized *RCAN1.2* antibody was not available at present, the protein expression experiment of *RCAN1.2* was not conducted. And additional invitro functional experiment focused on *RCAN1.2* was not carried out. Further study is needed to investigate the role of *RCAN1.2* in the development of ESCC by further assess the up- and down-stream signal pathway of *RCAN1.2*.

## Conclusions

To summarize, these findings show that *RCAN1* play a role in preventing proliferation, migration, and invasive activity of ESCC cells. And *RCAN1.2* mRNA of tumor tissue was identified as a novel prognostic marker in ESCC, targeting *RCAN1.2* may provide a potential therapeutic approach in ESCC.

## Supplementary Material

Supplementary tables.

## Figures and Tables

**Table 1:** Clinical characteristics of 100 ESCC patients according to *RCAN1.2* mRNA expression level

	*RCAN1.2* Low (n=50)	*RCAN1.2* High (n=50)	*P* value
**Age/years**			0.509
>60	34	37	
≤60	16	13	
**Gender**			0.023*
Female	13	24	
Male	37	26	
**Smoke**			0.069
Yes	26	17	
No	24	33	
**Drink**			0.105
Yes	25	17	
No	25	33	
**Family history of cancer**			1.000
Yes	15	15	
No	35	35	
**cardiovascular and cerebrovascular diseases**			0.548
Yes	28	25	
No	22	25	
**Stage**			0.032*
I-II	29	39	
III-IV	21	11	
**LNM**			0.008**
No	24	37	
Yes	26	13	
**Tumor diameter**			0.052
≥5cm	20	11	
<5cm	30	39	
**Differentiation**			0.656
Well	15	13	
Poor-Moderate	35	37	

**Footnote:** *P<0.05, **P<0.001

**Table 2:** Univariate and multivariate Cox analysis of factors for the overall survival of esophageal cancer patients

Variables	Univariate analysis		Multivariate analysis	
	HR	P value	HR	P value
Gender (Female vs. Male)	0.7487	0.329		
Age (≤60 vs >60)	0.5523	0.081	0.6483	0.21235
Smoke (Yes vs. No)	1.5616	0.111		
Drink (Yes vs. No)	1.01460	0.959		
Family history of cancer (Yes vs. No)	0.7379	0.332		
Cardiovascular and cerebrovascular diseases (Yes vs. No)	0.8189	0.476		
*RCAN1.1* (High vs. Low)	0.8032	0.433		
*RCAN1.2* (High vs. Low)	0.4669	0.00848**	0.5266	0.03554*
*RCAN1.4* (High vs. Low)	0.8344	0.515		
LNM (Yes vs. No)	3.2713	2.76E-05***	4.1575	0.00112**
Stage (I-II vs III-IV)	0.4358	0.00312**	1.7347	0.20912
Tumor diameter (≥5cm vs <5cm)	1.2610	0.426		
Differentiation (Well vs Poor-moderate)	0.7519	0.363		

**Footnote:** *P<0.05, **P<0.001, ***P<0.001

## Abbreviations

EC: Esophageal cancer
ESCC: esophageal squamous cell carcinoma
LNM: lymph node metastasis
OSCC: oral squamous cell carcinomas
OS: overall survival
