# Supplementary material for: Low RCAN1.2 mRNA Expression Is Associated with Poor Prognosis of Patients with Esophageal Squamous Cell Carcinoma
Source: J Cancer. 2023 Jul 31;14(12):2361–72. doi: 10.7150/jca.84307 (PMC10414045; doi:10.7150/jca.84307)
Supplement: Supplementary file 1 — Supplementary tables. [file jcav14p2361s1.pdf]

Supplementary Materials

Low *RCAN1.2* mRNA Expression Is Associated with Poor Prognosis of Patients with Esophageal Squamous Cell Carcinoma

Haijun Yang[1, 2#], Jiahuan Zhou[3#], Keyao He[4#], Junkuo Li[1,2], Fang Zhao[1,2], Ningtao Dai[1,2], Shouxin Wu[3], Wushuang Li[3], Jiangman Zhao[3*], Yaowen Zhang[1, 2*], Fuyou Zhou[1, 2*]

***Correspondence:** Jiangman Zhao, zhaojiangman86@163.com; Yaowen Zhang, zhangyaowen621@126.com; Fuyou Zhou, ayzhoufuyou@163.com

Supplementary Tables

Table S1 Primer sequences used for real-time PCR

	Forward primer	Reverse primer
RCAN1.1	3’-TGGAGCTTCATTGACTGCGA-3’	3’-ACGTCCTAAAGAGGGACTCA-3’
RCAN1.2	3’-GGCGACGTGACTCAGTGTTC-3’	3’-GGTGATGTCCTTGTCATACGTC-3’
RCAN1.4	3’-TTTAGCTCCCTGATTGCCTGT-3’	3’-AAAGGTGATGTCCTTGTCATACG-3’
GAPDH	3’-GCCACATCGCTCAGACACC-3’	3’-GATGGCAACAATATCCACTTTACC-3’

Table S2 The mRNA expression level of three *RCAN1* transcripts in ESCC tumor tissues compared with matched normal tissues in GSE164158 and GSE149609 datasets.

***RCAN1* transcripts**	**Dataset**	**Fold Change**	***P* value**	***P* adj**	**Regulated**
RCAN1.1	GSE164158	0.69	0.293398	0.583475	Down regulation
RCAN1.1	GSE149609	2.63	0.080205	0.325335	Up regulation
RCAN1.2	GSE164158	0.59	0.248591	0.535982	Down regulation
RCAN1.2	GSE149609	0.39	0.464981	0.777611	Down regulation
RCAN1.4	GSE164158	0.79	0.667443	0.85669	Down regulation
RCAN1.4	GSE149609	1.88	0.132468	0.428839	Up regulation

Table S3 Clinical characteristics of 100 ESCC patients according to *RCAN1.1* mRNA expression level

	***RCAN1.1* Low (n=50)**	***RCAN1.1* High (n=50)**	***P* value**
**Age/years**			0.015*
>60	30	41	
⩽60	20	9	
**Gender**			0.534
Female	17	20	
Male	33	30	
**Smoke**			0.545
Yes	20	23	
No	30	27	
**Drink**			0.224
Yes	24	18	
No	26	32	
**Family history of cancer**			0.383
Yes	17	13	
No	33	37	
**cardiovascular and cerebrovascular diseases**			0.841
Yes	26	27	
No	24	23	
**Stage**			0.198
I-II	31	37	
III-IV	19	23	
**LNM**			0.539
No	29	32	
Yes	21	18	
**Tumor diameter**			0.517
⩾5cm	14	17	
<5cm	36	33	

**Differentiation**			0.373
Well	16	12	
Poor-Moderate	34	38	

Footnote: $^*P<0.05$

Table S4 Clinical characteristics of 100 ESCC patients according to *RCAN1.4* mRNA expression level

	***RCAN1.4* Low (n=50)**	***RCAN1.4* High (n=50)**	***P* value**
**Age/years**			0.015*
>60	30	41	
⩽60	20	9	
**Gender**			0.147
Female	15	22	
Male	35	28	
**Smoke**			0.313
Yes	24	19	
No	26	31	
**Drink**			0.015*
Yes	27	15	
No	23	35	
**Family history of cancer**			1.000
Yes	15	15	
No	35	35	
**cardiovascular and cerebrovascular diseases**			0.316
Yes	29	24	
No	21	26	
**Stage**			0.198
I-II	31	37	
III-IV	19	13	
**LNM**			0.539
No	29	32	
Yes	21	18	
**Tumor diameter**			0.517
⩾5cm	17	14	
<5cm	33	36	

**Differentiation**			0.026*
Well	9	19	
Poor-Moderate	41	31	

Footnote: $^{*}P<0.05$

Table S5 Univariate and multivariate Cox analysis of factors for the overall survival of 87 esophageal cancer patients after PSM analysis.

Variables	Univariate analysis		Multivariate analysis	
	HR	P value	HR	P value
Gender (Female vs. Male)	0.7561	0.36		
Age (≤60 vs >60)	0.5155	0.0621	0.6119	0.17609
Smoke (Yes vs. No)	1.5096	0.152		
Drink (Yes vs. No)	1.01032	0.972		
Family history of cancer (Yes vs. No)	0.7397	0.365		
Cardiovascular and cerebrovascular diseases (Yes vs. No)	0.7565	0.332		
RCAN1.1 (High vs. Low)	0.8611	0.603		
RCAN1.2 (High vs. Low)	0.5331	0.0398*	0.5446	0.05737
RCAN1.4 (High vs. Low)	0.8874	0.677		
LNM (Yes vs. No)	3.0092	0.000194***	3.9346	0.00272**
Stage (I-II vs III-IV)	0.4506	0.00575**	1.6836	0.24633
Tumor diameter (≥5cm vs <5cm)	0.6509	0.175		
Differentiation (Well vs Poor-moderate)	1.2765	0.41		

Footnote: **P*<0.05, ***P*<0.001, ****P*<0.001
